# Healing of Ischemic Colon Anastomosis in Rats Could Be Provided by Administering Dexpanthenol or Coenzyme Q10

**DOI:** 10.3390/jcm7070161

**Published:** 2018-06-25

**Authors:** Faruk Pehlivanlı, Oktay Aydin, Gökhan Karaca, Gülçin Aydin, Tuba Devrim, Huri Bulut, Bülent Bakar, Çağatay Erden Daphan

**Affiliations:** 1 Department of General Surgery, Kirikkale University School of Medicine, Kirikkale 71450, Turkey; drfapeh@hotmail.com (F.P.); gokhankaracaa@yahoo.com (G.K.); cagatayerden@yahoo.com (Ç.E.D.); 2 Department of Anesthesiology and Reanimation, Kirikkale University School of Medicine, Kirikkale 71450, Turkey; drgulcinaydin@yahoo.com; 3 Department of Pathology, Kirikkale University School of Medicine, Kirikkale 71450, Turkey; tubadevrim@gmail.com; 4 Department of Medical Biochemistry, Bezmiâlem Vakıf University School of Medicine, Istanbul 34093, Turkey; huribulut@gmail.com; 5 Department of Neurosurgery, Kirikkale University School of Medicine, Kirikkale 71450, Turkey; bulentbanrs@yahoo.com

**Keywords:** dexpanthenol, Coenzyme coenzyme Q10, anastomosis, colon, ischemia

## Abstract

Background: In this study, the effectiveness of dexpanthenol and coenzyme Q10 (CoQ10) on the healing of ischemic colon anastomosis was investigated. Methods: Forty eight male Wistar Albino rats were divided into four equal groups (Sham-S, Sham-I, DXP, Q10). Following full layer colon resection, single layer colon anastomosis, without creating ischemia, was performed on the Sham-S group. The same experimental model was performed on remaining groups after ischemia was created. Intraperitoneal dexpanthenol and CoQ10 was administered to the DXP and Q10 groups once a day for three days. Ten days later, all colon anastomoses were investigated histopathologically and biochemically, as well as their burst pressure values, in all sacrificed rats. Results: The highest burst pressure value was observed in the Sham-S group, decreasing from high to low in the DXP, Q10, and Sham-I groups, respectively (*p* = 0.008). Furthermore, tissue hydroxyproline (*p* = 0.001) level values were significantly different among the groups. Additionally, histopathological analysis revealed a significant difference among groups regarding reepithelization (*p* = 0.027) and polymorphonuclear leukocyte density (*p* = 0.022). Conclusions: This preliminary study has shown that ischemia-reperfusion injury may impair the healing of colon anastomosis and it has been concluded that dexpanthenol and CoQ10 may have positive effects on the healing of ischemic colon anastomosis in rat, although re-epithelization may be adversely affected using CoQ10.

## 1. Introduction

Ischemic colitis, classified into gangrenous and nongangrenous forms, is a common and, potentially, lethal disease of the gastrointestinal tract. Although ischemic colitis may develop spontaneously, it is usually present in advanced ages, arteriosclerosis, congestive heart failure, aortic surgery, vasculitis, cocaine and/or some other drug abuse, coagulopathies, and some medications (digitalisler, nasal decongestants, oral contraceptives, etc.). Approximately 14–66% of patients diagnosed with ischemic colitis require surgery (colon anastomoses after colectomies, etc.). It is argued that urgent operative intervention must be performed on gangrenous colonic ischemia due to the high morbidity and mortality rates [1,2]. However, the presence of ischemia, tension, and/or infection in the anastomotic region commonly causes disruptions of healing procedures and leads to anastomotic leaks after surgery performed on the ischemic colon [3]. On the other hand, the healing of wounds exposed to ischemia in colon anastomosis may be delayed, in particular by ischemia, and this also may lead to anastomotic leakage and/or infection [4]. Additionally, Benacerraf et al. [5,6] pointed out that the anastomosis leakage risk rate was almost 100% in cases where perianastomotic tissue oxygen pressure levels dropped below 20 mm-Hg, and the rate of anastomotic leakage decreased with increases in oxygen pressure [5,6]. Therefore, anastomotic leaks after intestinal, colonic, and colorectal anastomosis surgery continue to be an important problem today [7,8]. Although the number of studies aimed at preventing or reducing ischemic damage in colon anastomosis has increased in recent years, as far as we know, no research has been found in the literature investigating the effects of dexpanthenol or coenzyme Q10 (CoQ10) administration on the healing of ischemic colonic anastomosis [4]. Dexpanthenol is the biologically active alcohol of pantothenic acid. It can penetrate easily into the skin when administered locally, and its topical form has significant therapeutic effects on skin epithelization and granulation through its stimulating effects of pentatonic acid on anti-inflammatory and fibroblast proliferation [4,9,10,11,12]. It can also provide antioxidant effects through increasing glutathione, Coenzyme A, and adenosine triphosphate (ATP) synthesis, prevent tissue damage from ischemia, and contribute to wound healing [4,12]. On the other hand, CoQ10 is an essential element in the mitochondrial respiratory chain and is synthesized endogenously. This fat-soluble material has anti-inflammatory and antioxidant properties [13,14,15]. It protects the membrane phospholipids and proteins from lipid peroxidation by scavenging free radicals directly and/or regenerating tocopherol levels. CoQ10 regulates mitochondrial permeability transition pores and the activation of the mitochondrial uncoupling proteins and inhibits lipid peroxidation in mitochondria, protein oxidation, and DNA oxidation. It also has potential redox activity in both the Golgi apparatus and lysosomes, and regulates the NADH oxido-reductase activity in the plasma membrane. Additionally, CoQ10 can affect apoptotic events, such as ATP depletion, release of cytochrome c into the cytosol, caspase-9 activation, depolarization of the mitochondrial membrane potential, and DNA fragmentation [16,17].

Therefore, this experimental pilot study is conducted to investigate the effects of these pharmacological agents on the healing of ischemic colon anastomosis.

## 2. Materials and Methods

### 2.1. Materials

Local Animal Care and Use Committee approval was obtained for this study (Decision number: 2015-15/10; approval date: 25 February 2015).

Forty-eight male Wistar Albino rats weighing 250–275 gr were divided randomly into four equal groups, as following (*n* = 12):

- Sham-S group: A full layer colon resection was performed without creating ischemia to the rats, and end to tip anastomosis was applied without experimental drug administration. The rats were sacrificed at the end of 10 days.
- Sham-I group: After ischemia was performed on the colon, a full layer colonic resection was performed and end to tip anastomosis was applied without experimental drug administration. The rats were sacrificed at the end of 10 days.
- DXP group: After ischemia was performed on the colon, a full layer colonic resection was performed and end to tip anastomosis was applied. Dexpanthenol (Bepanthene 500 mg ampoule, Roche, Berlin, Germany) was administered at a dose of 50 mg/kg once a day for three days, intraperitoneally, and rats were sacrificed at the end of 10 days.
- Q10 group: After ischemia was performed on the colon, a full layer colonic resection was performed and end to tip anastomosis was applied. CoQ10 (Sigma Chemical Co., St. Louis, MO, USA) was administered at a dose of 1 mg/100g once a day for three days, intraperitoneally, and rats were sacrificed at the end of 10 days.

Sedation anesthesia for the surgical procedures was applied to all rats by administering 10 mg/kg of ketamine (Ketalar^®^, Pfizer, New York, NY, USA) and 90 mg/kg of xylazine (Rompun^®^, Bayer, Leverkusen, Germany) through the intramuscular route.

### 2.2. Surgery

After sedation by anesthesia was applied to all animals, the abdominal wall was opened with a midline laparotomy and the superior mesenteric artery was occluded using a clamp (Halstead-mosquito clamp, flat, BH-110, 12.5 cm) for 20 min, excluding those of the Sham-S group. After removal of the clamp, the artery and tissues were reperfused. Following the full layer colon resection (about 5 cm distal to the cecum), performed without damaging the mesocolon, colon anastomosis was performed end to tip using a single layer 6/0 round-tipped needle prolene suture (Prolene^®^, Ethicon, Somerville, NJ, USA) along the incision [18,19]. Then, the experimental pharmacologic agents were injected into the animals of the study groups intraperitoneally, as described above. Ten days later, all animals were subjected to re-laparotomy under anesthesia and the anastomosis line was removed from 2 cm proximal and 2 cm distal of this line. Then, the rats were sacrificed with a high dose anesthetic agent after a blood sample was taken from the portal vein. The burst pressure was measured at the anastomosis line of the removed colon and, after this procedure, the tissue covering the anastomosis line was divided into two equal parts. The part to be taken for histopathological evaluation was stored in formaldehyde, and the other part, which was reserved for biochemical analysis, was placed in a deep freezer at a temperature of −40 °C in a physiological saline solution. The venous blood samples were stored in a deep freezer at −40 °C after centrifugation.

### 2.3. Burst Pressure Measurement

The distal and proximal ends of the colon segment, taken in a previously prepared room that was insulated against external factors at a normal room temperature (20 ± 2 °C), were placed and connected to measure the bursting pressure in the anastomosis line of the removed colon tissue [20,21]. A physiological saline solution was pumped into the system at a constant flow (1.5 mL/min) with an infusion pump (BIOPAC Systems Inc., Santa Barbara, CA, USA), and the resulting pressure changes and burst pressure data were recorded electronically (MP30 data Acquisition System, Santa Barbara, CA, USA). The obtained data were evaluated using the MP30 data analysis system.

### 2.4. Biochemical Analysis

Tissue samples weighing 1 gr, which were retained for biochemical analysis, were extracted after the application of pure HCL at the 1200 °C temperature (high temperature). After centrifugation of the obtained extract at 5000 rpm for 30 min, the absorbance of the material was evaluated colorimetrically (photometric) at 121 °C at 562 nm and the tissue hydroxyproline (HP) and serum HP levels (Rat Hydroxyproline Elisa Kit: Yehua Biological Technology Co Ltd., Shanghai, China) were measured using the enzyme linked immunoabsorbent assay (ELISA).

### 2.5. Histopathological Examination

**Figure 1:**
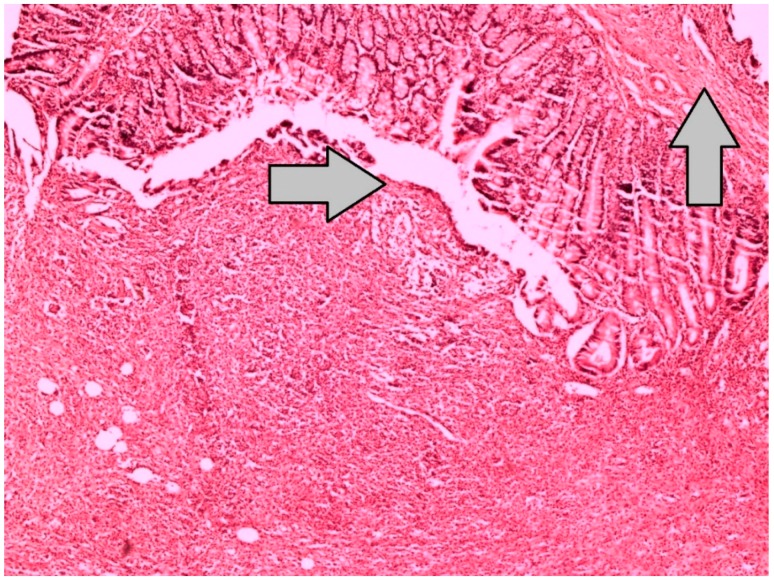
This specimen, which represents the grade 0 reepithelization at the anastomotic line of the Sham-I group, reveals dense inflammation, with neutrophil-rich leukocyte infiltration and without epithelization in the line of anastomosis and the stroma (H&E × 100).

**Figure 2:**
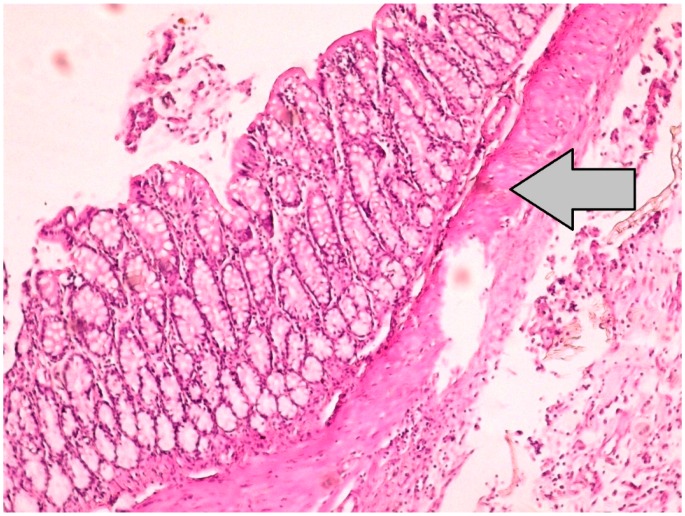
Histopathological image, which represents the grade 3 reepithelization at the anastomotic line of the Sham-S group, demonstrates the large intestinal mucosa, with the epithelial layer precisely seen on the surface of the colon anastomosis (H&E × 100).

**Figure 3:**
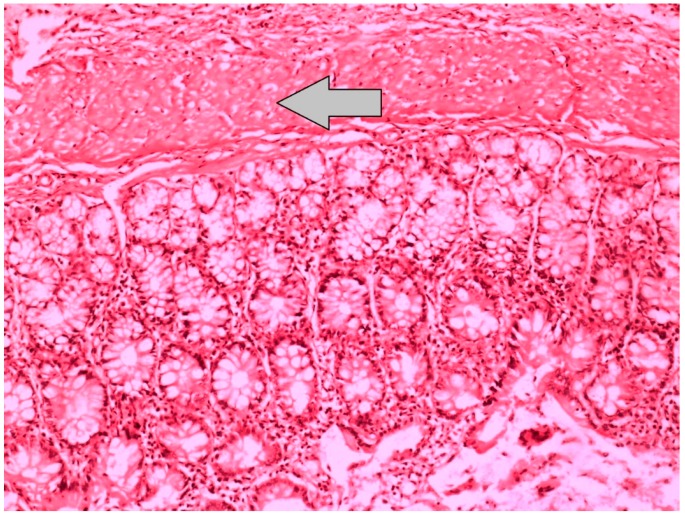
Histopathological image, which represents the grade 1 inflammation at the anastomotic line of the Sham-S group, demonstrates the intestinal mucosa, with no neutrophil infiltration and regular appearance of crypt structures and muscularis propria (H&E × 100).

**Figure 4:**
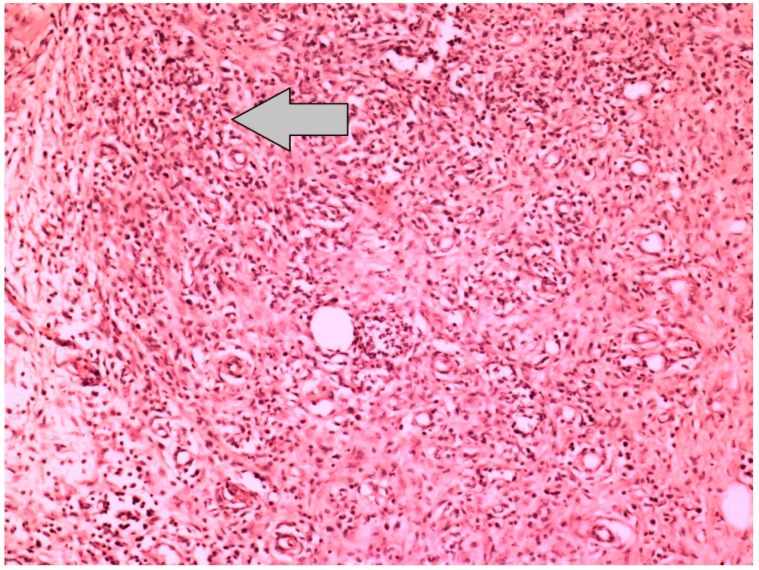
Histopathological image, which represents the grade 4 inflammation at the anastomotic line of the Sham-I group, shows the neutrophil-rich dense inflammatory cell infiltration and capillary vessel proliferation in the colon anastomosis region (H&E × 100).

After tissues were embedded in paraffin blocks, serial sections that were retained for histopathological examination were stained with hematoxylin and eosin (H&E), and the colon tissue along the anastomosis line was evaluated for reepithelization, neovascularization, polymorphonuclear leukocyte (PMNL) density, lymphocyte count, and completeness of the muscular layer under the light microscope (U-MDOB3, Olympus, Tokyo, Japan) by a pathologist who was blinded to the study and study groups (Table 1, Figure 1, Figure 2, Figure 3 and Figure 4) [8,22].

### 2.6. Statistical Analysis

The findings, which were distributed normally among groups, were tested by using the One-Way Analysis of Variance (ANOVA) test (*p* < 0.05). Post hoc evaluation was performed by using the Tukey Multiple Comparisons test (*p* < 0.05).

The values, which were not normally distributed among the acute and chronic period groups, were analysed by using the Kruskal-Wallis test (*p* < 0.05). For binary comparisons, the Mann-Whitney U test with the Bonferroni Correction test was performed (*p* < 0.0083).

## 3. Results

### 3.1. Burst Pressure

**Figure 5:**
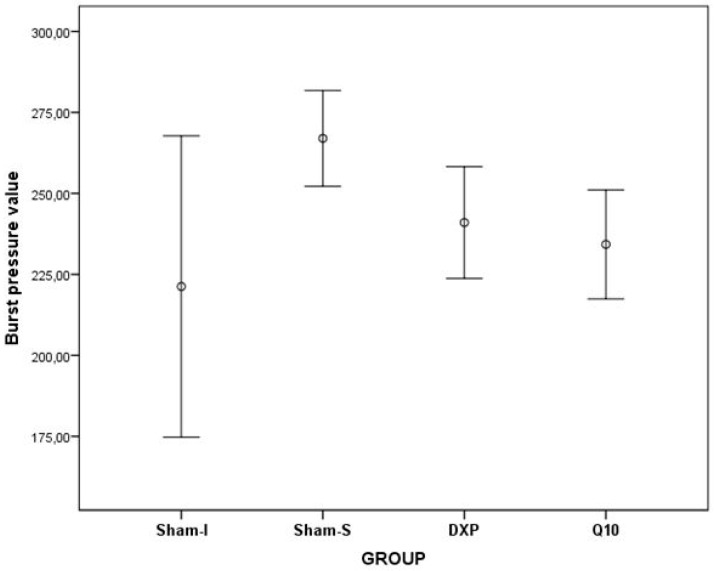
Error bars represent the burst pressure values of all groups.

The highest burst pressure was obtained in the non-ischemic Sham-S group, decreasing from high to low in the DXP group, the Q10 group, and the Sham-I group, respectively (*p* = 0.008) (Table 2 and Table 3, Figure 5).

In the binary comparison, the burst pressure values of the Sham-I group were lower than in the Sham-S group (*p* = 0.006), DXP group (*p* = 0.016), and Q10 group (*p* = 0.005), respectively. Although the burst pressure values of the DXP and Q10 groups were lower than the Sham-S group, numerically, these values were not different statistically. In addition, burst pressure values were not different between the DXP and Q10 groups, statistically (Table 4).

### 3.2. Biochemical Analysis

**Figure 6:**
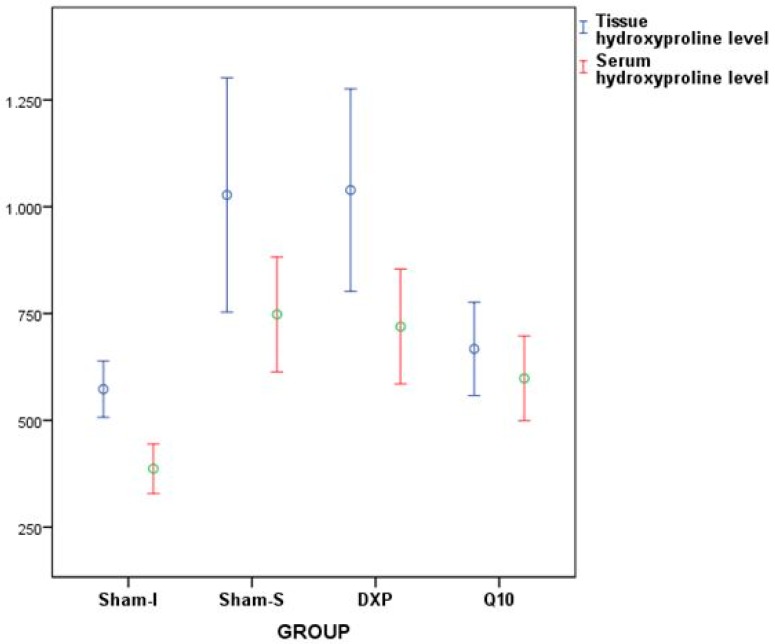
Error bars represent the tissue and serum hydroxyproline level values of all groups.

According to biochemical data, the tissue HP (*p* = 0.001) and serum HP (*p* <0.001) values were significantly different among the groups (Table 2 and Table 3, Figure 6).

Binary comparison results showed that the tissue HP level values were different between the Sham-S and Sham-I groups (*p* = 0.003), between the Sham-I and DXP groups (*p* = 0.002), between Sham-S and Q10 groups (*p* = 0.020), and between DXP and Q10 groups (*p* = 0.015). However, serum HP level values were found to be different between the Sham-S and Sham-I groups (*p* < 0.001), between the Sham-I and DXP groups (*p* < 0.001), and between the Sham-I and Q10 groups (*p* = 0.018) (Table 4).

### 3.3. Histopathological Evaluation

**Figure 7:**
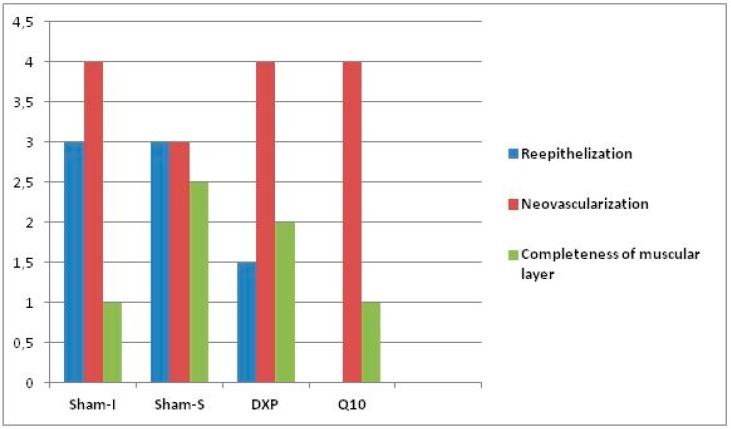
Bars represent the reepithelization, neovascularization, and muscular layer completeness score values.

**Figure 8:**
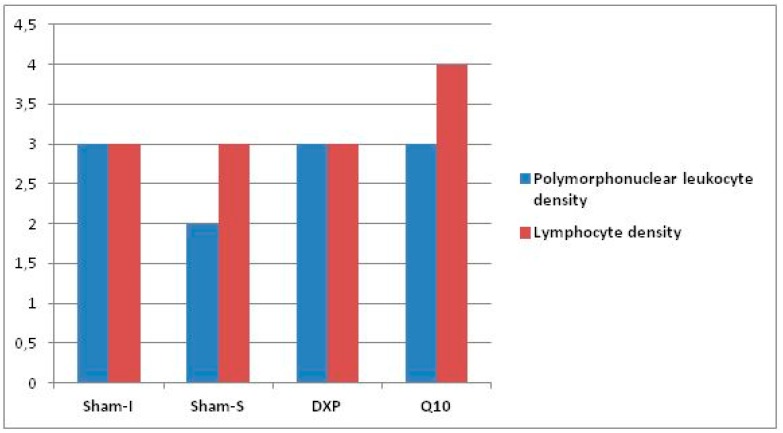
Bars represent the polymorphonuclear leukocyte density and lymphocyte density score values.

The histopathological data revealed a significant difference among the groups regarding reepithelization (*p* = 0.027) and PMNL density (*p* = 0.022). However, no difference was observed regarding neovascularization, lymphocyte count, and muscular layer completeness (Table 5 and Table 6, Figure 7 and Figure 8).

Binary comparison results showed that the reepithelization level values were different between the Sham-S group and Q10 group (*p* = 0.003), while the PMNL concentration level values of the Sham-I group were higher than the Sham-S group values (*p* = 0.003) (Table 7).

## 4. Discussion

In fact, burst pressure is used to show mechanical resistance and force in the colon anastomosis line, whereas HP levels are used to identify the tissue resistance and collagen content of the peri-anastomotic region, indirectly [5,9,20,23]. In this study, burst pressure level values were observed to be significantly higher in the non-ischemic Sham-S group than in the ischemia induced groups. This finding suggested that ischemia negatively affected the healing of colon anastomosis, and this result supported the data, reflecting that oxidative stress disrupts wound healing as discussed in the literature [24]. However, burst pressure values of the DXP group were similar to the Sham-S group values, while being statistically different from the Sham-I group values. In this regard, dexpanthenol use may have positive effects on the healing of ischemic colon anastomosis regarding burst pressure values. The burst pressure values of the Q10 group were also observed to be higher, but it was not as high as in the Sham-S and DXP groups. On the other hand, tissue HP level values were observed to be significantly higher in the Sham-S group than in the Sham-I group. These results supported the suggestion that ischemia has a distorting/reducing effect on wound healing along the anastomotic line of the colon.

In this study, tissue HP level values were parallel to the burst pressure values. The tissue HP level values of the DXP group were found to be similar to the results of the Sham-S group, while being significantly higher than those values of the Sham-I and Q10 groups. However, the tissue HP level values of the Q10 group were observed to be numerically higher than the Sham-I group, but not to a statistically significant degree. These finding suggested that dexpanthenol could have positive effects on the healing of ischemic colon anastomosis in rats. Kitamura et al. showed that CoQ10 has positive effects on wound healing by suppressing oxidative stress and inflammatory reactions [25,26]. However, present study results demonstrated that CoQ10 had no therapeutic effect on the healing of ischemic colon anastomosis biochemically, although it could increase burst pressure values in the ischemic colon anastomosis line. With these results, dexpanthenol could make a meaningful positive contribution to the recovery of ischemic colon anastomosis and is promising for potential clinical use. Furthermore, serum HP levels of the DXP group supported these findings. In conclusion, according to these findings, ischemia can disrupt the recovery of colon anastomosis, in terms of both biochemical and mechanical strength, in rats. It was thought that the use of dexpanthenol and coenzyme Q10 in rats with ischemic colon anastomosis could have positive effects on improving ischemic colon anastomosis, especially in the use of dexpanthenol.

Although positive effects of CoQ10 on ischemic wound healing have been reported in previous studies, there is limited data about the effects of CoQ10 on reepithelization in the literature [14,15]. Ebner et al. reported that the administration of dexpanthenol can accelerate reepithelization and fibroblast proliferation in wound healing by inducing epithelization and granulation formation in epidermal renewal [26,27]. In the present study, the reepithelization level was observed to be significantly higher in the Sham-S group than in the DXP and Q10 groups. Therefore, it was thought that neither dexpanthenol nor CoQ10 had a therapeutic effect on the reepithelization of the ischemic colon anastomosis line. Although CoQ10 administration after ischemia was observed to have a minor positive contribution regarding tissue HP levels and burst pressure values, it was thought to have a negative effect on wound healing regarding reepithelization [9]. Concurring with the literature, which concluded that PMNL levels increase in ischemic tissue after ischemia-reperfusion injury, it was found in the present study that the highest tissue PMNL density was measured in the Sham-I group. PMNL density slightly decreased in the groups that received dexpanthenol and CoQ10 when compared to the Sham-I group, but this decrease was not statistically significant. With these results, it was thought that both agents had some detractive effects on PMNL density in the wound healing process due to their anti-inflammatory and antioxidant properties [14,15,28,29]. However, any experimental agent did not affect the neovascularization and muscular layer completeness grade values. Overall, it was concluded from these result that neither dexpanthenol nor CoQ10 has therapeutic effects on ischemic colon anastomosis in rats, histopathologically.

## 5. Limitations

This preliminary study contained some limitations. First, an altered metabolism, infarcted tissue volume, and anastomotic leakage in ischemic colon anastomosis was not demonstrated by using radiodiagnostic methods. Second, due to financial restrictions, the anastomotic colonic tissue was not examined immunohistochemically and ultrastructurally. Moreover, specific biochemistry techniques were not performed to detail the effects of the experimental agents to other inflammatory cascades. Third, although the effects of dexpanthenol and coenzyme Q10, especially on ischemic colonic anastomosis, were investigated in this study, the effects of these agents on non-ischemic colonic anastomosis should be investigated in further studies. Fourth, local administration of these agents was not performed on the rats to investigate its local effects in ischemic colon anastomosis. Furthermore, an experimental group to which both agents were administered should be included in the study. For these reasons mentioned above, it can be recommended, with those unexpected findings of this preliminary study, that various dosages with different administration methods and time protocols of these pharmacological agents in ischemic or non-ischemic colon anastomosis should be detailed and tested in further studies.

## 6. Conclusions

This preliminary study has shown that ischemia-reperfusion injury may impair the healing of colon anastomosis and it has been concluded that dexpanthenol and CoQ10 may have positive effects on the healing of ischemic colon anastomosis in rats, although re-epithelization may be adversely affected using CoQ10.

## Figures and Tables

**Table 1:** Descriptive table of the histopathological grading scores [8].

**Reepithelization of Anastomotic Mucosa (Components)**	
Grade 0	No epithelization at anastomotic line
Grade 1	Incomplete coverage of the anastomotic line with a single cell layer
Grade 2	Complete coverage of the anastomotic line with a single cell layer
Grade 3	Complete reepithelization with glandular epithelium
**Neovascularization**	
Grade 1	None
Grade 2	Minimal
Grade 3	Moderate
Grade 4	Severe
**Inflammation at Anastomotic Line (Polymorphonuclear Leukocyte or Lymphocyte)**	
Grade 1	None
Grade 2	Minimal
Grade 3	Moderate
Grade 4	Severe
**Completeness of Muscular Layer**	
Grade 1	Complete disruption
Grade 2	Incomplete disruption
Grade 3	Complete healing

**Table 2:** Descriptive table of the burst pressure values and biochemical analysis results of all groups (SD: Standard deviation).

Group	Variable	Minimum	Maximum	Mean	SD
Sham-I	Burst pressure	131.00	260.00	221.25	55.65
	Tissue hydroxyproline level	446.37	623.99	573.06	78.92
	Serum hydroxyproline level	315.61	492.89	386.95	69.65
Sham-S	Burst pressure	250.00	305.00	267.00	17.67
	Tissue hydroxyproline level	485.12	1466.74	1027.66	328.06
	Serum hydroxyproline level	386.27	892.65	747.96	161.27
DXP	Burst pressure	208.00	277.00	241.00	20.63
	Tissue hydroxyproline level	653.27	1434.89	1039.09	283.23
	Serum hydroxyproline level	354.42	860.80	719.54	161.05
Q10	Burst pressure	201.00	270.00	234.25	20.10
	Tissue hydroxyproline level	511.58	831.83	667.26	130.49
	Serum hydroxyproline level	469.58	830.66	598.36	118.75

**Table 3:** This table demonstrates the differences of the burst pressure level values and biochemical analysis results among the groups. The One-Way Analysis of Variance (ANOVA) test *p* < 0.05 (*F*: *F* score).

Variable	*F*	*p*
Burst pressure value	6.797	0.008
Tissue hydroxyproline level	8.855	<0.001
Serum hydroxyproline level	12.168	<0.001

**Table 4:** The post hoc test results of the burst pressure level values and biochemical analysis findings. The Tukey Multiple Comparisons test, *p* < 0.05 (HP: Hydroxyproline).

Group (I/J)	Burst Pressure	Tissue HP Level	Serum HP Level
Sham-I/Sham-S	0.006	0.003	<0.001
Sham-I/DXP	0.016	0.002	<0.001
Sham-I/Q10	0.005	0.844	0.018
Sham-S/DXP	0.752	1.000	0.973
Sham-S/Q10	0.371	0.020	0.135
DXP/Q10	0.343	0.015	0.285

**Table 5:** Descriptive table of the histopathological grade values of all groups (Min: Minimum, Max: Maximum, SD: Standard deviation).

Group	Variable	Min	Max	Median	SD
Sham-I	Reepithelization	0	3	3	1.41
	Neovascularization	3	4	4	0.35
	Polymorphonuclear leukocyte density	3	4	3	0.35
	Lymphocyte density	3	4	3	0.53
	Muscular layer completeness	1	2	1	0.51
Sham-S	Reepithelization	3	3	3	0.00
	Neovascularization	2	4	3	0.75
	Polymorphonuclear leukocyte density	2	3	2	0.46
	Lymphocyte density	3	4	3	0.46
	Muscular layer completeness	1	3	2.50	0.88
DXP	Reepithelization	0	3	1.50	1.60
	Neovascularization	2	4	4	0.74
	Polymorphonuclear leukocyte density	1	3	3	0.75
	Lymphocyte density	2	4	3	0.64
	Muscular layer completeness	1	3	2	0.83
Q10	Reepithelization	0	3	0	1.41
	Neovascularization	3	4	4	0.51
	Polymorphonuclear leukocyte density	2	3	3	0.51
	Lymphocyte density	3	4	4	0.51
	Muscular layer completeness	1	3	1	0.91

**Table 6:** This table demonstrates the differences in the histopathological grading score values among the groups. Kruskal-Wallis test *p* < 0.05 (*X*^2^: Chi-square).

Variable	*X* ^2^	*p*
Reepithelization	9.192	0.027
Neovascularization	7.577	0.056
Polymorphonuclear leukocyte density	9.616	0.022
Lymphocyte density	3.867	0.276
Muscular layer completeness	5.618	0.132

**Table 7:** The post hoc test results of the histopathological grading score values. The Mann-Whitney *U* test with the Bonferroni Correction, *p* < 0.0083. (PMNL: Polymorphonuclear leukocyte density).

Group	Reepithelization	PMNL
Sham-I/Sham-S	0.064	0.003
ShamI/DXP	0.473	0.045
Sham-I/Q10	0.135	0.044
Sham-S/DXP	0.025	0.289
Sham-S/Q10	0.003	0.143
DXP/Q10	0.473	0.854
